# Rapid identification of bacteria using a multiplex polymerase chain reaction system for acute abdominal infections

**DOI:** 10.3389/fmicb.2023.1220651

**Published:** 2023-07-10

**Authors:** Nanako Kakizaki, Koji Asai, Makoto Kuroda, Ryutaro Watanabe, Manabu Kujiraoka, Tsuyoshi Sekizuka, Miwa Katagiri, Hodaka Moriyama, Manabu Watanabe, Yoshihisa Saida

**Affiliations:** ^1^Department of Surgery, Toho University Ohashi Medical Center, Tokyo, Japan; ^2^Department of Clinical Oncology, Toho University Graduate School of Medicine, Tokyo, Japan; ^3^Laboratory of Bacterial Genomics, National Institute of Infectious Diseases, Pathogen Genomics Center, Tokyo, Japan

**Keywords:** acute abdominal infections, multiplex polymerase chain reaction system, metagenomic analysis, rapid identification, FilmArray system

## Abstract

**Purpose:**

Acute abdominal infections can be fatal if the causative organism (s) are misidentified. The spread of antimicrobial-resistant bacteria has become a serious problem worldwide, making antibiotic selection extremely difficult. Using quantitative metagenomic analysis, we evaluated a commercial multiplex polymerase chain reaction (PCR) system (FilmArray™, bioMérieux, Marcy-l’Étoile, France) for the rapid identification of causative bacteria.

**Methods:**

The cases of 10 patients with acute abdominal infections were enrolled in this retrospective study. There were six cases of perforated peritonitis and four cases of intraabdominal abscess. Fluid collected from the acute surgical abdominal infections were examined.

**Results:**

All specimens tested positive for microorganisms in culture, and six involved two or more microorganisms. Using the multiplex PCR system, nine of ten specimens were found to involve at least one microorganism. One specimen was not included in the multiplex PCR system panel. Nineteen of 21 microorganisms (90.5%) isolated by culture were detected by the multiplex PCR system. Microorganisms with very small numbers of reads (19 reads) were detectable.

**Conclusion:**

This multiplex PCR system showed a high detection rate for causative microorganisms in ascites and intraabdominal abscesses. This system may be suitable as an affordable rapid identification system for causative bacteria in these cases.

## 1. Introduction

Acute abdominal infections such as perforated peritonitis and intraabdominal abscesses can be fatal if inappropriate antimicrobial therapy is employed. In addition, the spread of antimicrobial-resistant (AMR) bacteria is now a serious problem worldwide, making antimicrobial selection extremely difficult (Thompson, 2022). In 2019, the aggregate number of deaths due to infection by AMR worldwide was reported to be 4.95 million. Of these, 1.27 million deaths were reported as due to direct AMR infection (Thompson, 2022). One reason for this increase in the incidence of AMR is the overuse of broad-spectrum antimicrobial agents. In bacterial culture testing, it takes approximately 5 days to completely identify the causative bacteria and provide antimicrobial susceptibility results (Pardo et al., 2016). Therefore, severe cases often require the use of broad-spectrum antimicrobial agents. This clinical situation highlights the urgent need for research on rapid identification of causative organisms for the selection of the appropriate narrower-spectrum antimicrobial agent(s).

In our previous study, we focused on acute biliary tract infection and performed comprehensive bacterial identification using bile specimens with metagenomic analysis (Kujiraoka et al., 2017). This method enables the identification of potential causative bacteria and AMR genes within 2 days. However, the complicated testing procedures and high testing costs hamper the use of metagenomic analysis with high patient numbers in clinical practice. Next, we performed a rapid bacterial identification analysis of bile specimens from acute biliary tract infection using the Verigene system (Luminex Corporation, Austin, TX, United States, previously Nanosphere, United States) (Watanabe et al., 2021). This rapid bacterial identification system was able to identify the causative bacteria within 2 h of examination. However, the detection rate of culture-positive bile specimens by Verigene system was only 35.7%, which is problematic for clinical practice. Another problem is that Gram staining is required to be performed prior to the test because the test panels of the Verigene system differ for Gram-positive and-negative bacteria.

In this study, we examined a new multiplex polymerase chain reaction (PCR) system (FilmArray™, bioMérieux, Marcy-l’Étoile, France), blood culture identification (BCID) panel. We did this by evaluating specimens collected from patients with suspected acute abdominal infections using the multiplex PCR system, and evaluated the results quantitatively using metagenomic analysis.

## 2. Methods

### 2.1. Patients

Ten patients with acute abdominal infection treated at Toho University Ohashi Medical Center from April 2019 to December 2019 were enrolled in this study. The protocol of this study was approved by the Ethics Committee of our hospital (approval number: H21090_H17077) and the National Institute of Infectious Diseases (approval number: 722). Written informed consent was obtained from patients before specimen analysis.

### 2.2. Sample collection

Intraabdominal samples were aseptically collected intraoperatively in six cases and via a drainage tube in four cases. Each specimen was divided into three anaerobic porters (approximately 2–3 mL each); one of these was submitted to the in-hospital bacteriology laboratory for culture identification and antimicrobial susceptibility testing, and the other two were frozen at −20°C and transported to the Laboratory of Bacterial Genomics, Pathogen Genomics Center, National Institute of Infectious Diseases, Tokyo, Japan for evaluation using the multiplex PCR system and metagenomic analysis, respectively.

### 2.3. Antimicrobial susceptibility testing

Antimicrobial susceptibility testing was performed (Neg EN Combo 1 T® panel, Microscan Walkaway 96SI: Siemens, Erlangen, Germany) according to the criteria of the Clinical and Laboratory Standards Institute (M100-S26).

### 2.4. Multiplex PCR system analysis

This system is a molecular diagnostic method based on multiplex PCRs performed on culture-positive blood. The multiplex PCR system BCID panel is a rapid bacterial identification system used in bloodstream infections that can evaluate 24 micro-organisms (8 Gram-positive bacteria, 11 Gram-negative bacteria, and 5 *Candida* spp.), as well as AMR genes (*mecA*, *vanA/B*, KPC) in 1 h (Supplementary Table S1). It is currently approved for testing blood samples; we used it for ascites/intraabdominal abscess fluid.

### 2.5. Metagenome analysis and bioinformatics

After pre-test preparation of clinical specimens, DNA-seq libraries were prepared using commercial kits (Illumina Nextra® XT DNA Sample Preparation Kit, Illumina; San Diego, CA, United States) for preparation and sequencing [NextSeq 500 reagents kit; sequencing runs of single-end short reads (150mer) NextSeq 500 sequencer, Illumina; San Diego, CA, United States]. To identify potential pathogens and detect AMR genes, sequence reads were analyzed using MePIC2 (Takeuchi et al., 2014), Krona (Ondov et al., 2011). Short read sequences for metagenomic analysis were deposited in the DNA Data Bank of Japan (Accession No. DRA005134).

## 3. Results

Specimens for examination using the multiplex PCR system were evaluated without any pre-test preparation. The multiplex PCR system BCID panel was able to test all 10 specimens at one time without software errors. Table 1 shows patient characteristics. Median age was 72 years (range: 57–97 years) and seven males and three females. There were six cases of perforated peritonitis and four cases of intraabdominal abscess.

**Table 1:** Patient characteristics and treatment.

Sample ID	Age	Sex	Diagnosis	Cause of abdominal infection	Specimen: abscess or ascites	Sampling method	Past medical history
**P1**	79	M	Intrabdominal abscess	Unidentified small intestine perforation	Abscess	Percutaneous abscess drainage	Thoracic aortic aneurysm
**P2**	79	F	Perforated peritonitis	Duodenal ulcer perforation	Ascites	Intraoperative	Tervical spondylosis
**P3**	62	M	Perforated peritonitis	Jejunal perforation by double-balloon endoscopy	Ascites	Intraoperative	Alcoholic pacreatitis duodenal ulcer
**P4**	97	F	Perforated peritonitis	Ileum perforation by fish bone	Ascites	Intraoperative	Diabetes cerebral infarction
**P5**	71	M	Intrabdominal abscess	Biliary fistula after pancreaticoduodenectomy	Abscess	Sampling from drainage tube	Bile duct cancer
**P6**	73	M	Intrabdominal abscess	Pancreatic fistula after distal pancreatectomy	Abscess	Sampling from drainage tube	Pancreatic tail cancer diabetes
**P7**	83	M	Perforated peritonitis	Jejunum perforation by metastatic tumor rapture	Ascites	Intraoperative	Undifferentiated pleomorphic sarcoma
**P8**	71	M	Intrabdominal abscess	Anastomotic leakage of left hemi-colectomy	Abscess	Sampling from drainage tube	Ascending colon cancer
**P9**	58	F	Perforated peritonitis	Sigmoid colon perforation by ischemic colitis	Ascites	Intraoperative	Diverticulitis
**P10**	57	M	Perforated peritonitis	Gallbladder perforation by acute cholecystitis	Ascites	Intraoperative	Diabetes alcoholic cirrhosis

### 3.1. Identified microorganisms by culture and the multiplex PCR system

All specimens were positive for microorganisms in culture, and six were positive for two or more microorganisms. Using the multiplex PCR system, nine of ten specimens were matched to at least one microorganism. One specimen (*Prevotella* sp.) was among the microorganisms not included in the multiplex PCR system BCID panel.

Table 2 summarizes the results of the culture and multiplex PCR system for each microorganism. In culture, 28 microorganisms of 23 species were confirmed from the 10 specimens. Twenty-one microorganisms of 16 species were included in the multiplex PCR system BCID panel. Seven species including *Bacteroides*, *Prevotella*, *and Fusobactrium*, etc. were not included in the multiplex PCR system BCID panel. Using multiplex PCR system, 19 of 28 isolated microorganisms were detected. When limited to the species that were included in the multiplex PCR system BCID panel, the detection rate was 90.5% (19/21). *Escherichia coli* (*E. coli*) was the only microorganism that was culture-positive but multiplex PCR system-negative. All other culture-positive microorganisms were detectable by the multiplex PCR system BCID panel. *Candida* spp., *Streptococcus*, and *Staphylococcus* were culture-negative but multiplex PCR system-positive.

**Table 2:** Microorganisms identified by culture and the multiplex PCR system.

Microorganism (*n*)		Culture/multiplex PCR system		
		Positive/positive	Positive/negative	Negative/positive
Including in the multiplex PCR system BCID panel^a^				
1.	*Enterococcus faecium* (2)	2	0	0
2.	*Enterococcus faecalis* (1)	1	0	0
3.	*Enterococcus avium* (1)	1	0	0
4.	*Staphylococcus* (1)	0	0	1
5.	*Staphylococcus aureus* (1)	1	0	0
6.	*Streptcoccus* (2)	0	0	2
7.	*Streptococcus anginosus* (2)	2	0	0
8.	*Escherichia coli* (3)	1	2	0
9.	*Klebsiella pneumonie* (3)	3	0	0
10.	*Klebsiella aerogenes* (1)	1	0	0
11.	*Citrobacter freundii* (3)	3	0	0
12.	*Serratia marcescens* (1)	1	0	0
13.	*Pseudomonas aeruginosa* (1)	1	0	0
14.	*Candida albicans* (5)	1	0	4
15.	*Candida glabrata* (2)	0	0	2
16.	*Candida tropicalis* (1)	1	0	0
Total		19	2	9
Not including in the multiplex PCR system BCID panel^a^				
1.	*Bacteroides fragilis* group (1)	–	1	–
2.	*Prevotella* sp. (1)	–	1	–
3.	*Prevotella/Porphyromonas* (1)	–	1	–
4.	*Fusobacterium nucleatum* (1)	–	1	–
5.	*Peptstreptococcus micro* (1)	–	1	–
6.	*Eubacterium* sp. (1)	–	1	–
7.	*Morganella morganii* (1)	–	1	–
Total			7	
Total	23	19	9	9

PCR, polymerase chain reaction; BCID, blood culture identification.

a See Supplementary Table S1 for a list of bacteria that can be detected by the multiplex PCR system BCID panel.

### 3.2. Metagenomic analysis

The results of metagenomic analyses are shown in Table 3. The DNA concentration of the specimens was proportional to the number of total reads. The human genome was excluded from the MEPIC2 software for microorganism detection. MePIC2 software identified DNAs derived from *Homo sapiens* and a wide variety of microorganisms, including bacteria indigenous to the oral and intestinal tracts.

**Table 3:** Results of metagenomic analysis.

Sample ID	Sample origin	DNA concentration (ng/μL)	Total reads	Human genome reads	Human genome (%)	Remaining reads after exclusion of human genome	Number of reads for *E. coli* (rate of total reads)
P1	Small intestine	49.0	17,154,163	16,985,393	99.0	168,770	178 (1.04 × 10^−5^)
P2	Duodenum	60.0	24,030,443	23,928,673	99.6	101,770	1 (4.16 × 10^−8^)
P3	Jejunum	25.0	15,064,268	14,758,401	98.0	305,867	141 (9.36 × 10^−6^)
P4	Ileum	33.8	13,208,642	13,120,565	99.3	88,077	15 (1.14 × 10^−6^)
P5	Bile duct	0.8	860,450	505,987	58.8	354,463	522 (6.07 × 10^−4^)
P6	Pancreas	1.3	915,292	14,028	1.5	901,264	18 (1.97 × 10^−5^)
P7^a^	Jejunum	10.9	9,829,707	9,082,885	92.4	746,822	95,875 (9.75 × 10^−3^)
P8	Left colon	0.1	9,645	9,331	96.7	314	0
P9	Left colon	27.5	23,918,080	23,401,541	97.8	516,539	21 (8.78 × 10^−7^)
P10	Gallbladder	27.2	18,710,915	18,626,399	99.5	84,516	12 (6.41 × 10^−7^)

a *E. coli* was detected in only P7 by the multiplex PCR system.

*E. coli* is a particularly important pathogen in intraabdominal infection. In this study, we focused on *E. coli* and evaluated the number of reads using metagenomic analysis. Including the results of this evaluation of *E. coli*, an overview of each patient is presented in Table 4. And as representative cases, details of P3, P6, and P7 are shown below.

**Table 4:** Results of culture and the multiplex PCR system with metagenomic analysis.

Sample ID	Culture-positive microorganism	Number of reads in the metagenomic analysis	Rate of reads in the metagenomic analysis	Multiplex PCR system-positive microorganism	Number of reads in the metagenomic analysis	Rate of reads in the metagenomic analysis
**P1**	*Streptococcus anginosus* (2+)	3,723	2.17 × 10^−4^	*Streptococcus*	6,796	3.96 × 10^−4^
**P1**	*E. coli* (small amount)	178	1.04 × 10^−5^			
**P2**	*Cadida albicans* (small amount)	1,905	7.93 × 10^−5^	*Candida albicans*	1,905	7.93 × 10^−5^
**P3**	*Enterococcus faecium* (1+)	63,060	4.19 × 10^−3^	*Enterococcus*	83,555	5.55 × 10^−3^
**P3**	*Klebsiella aerogenes* (1+)	23,065	1.53 × 10^−3^	*Enterobacteriaceae*	25,387	1.69 × 10^−3^
**P3**	*Citrobacter freundii* (small amount)	57	3.78 × 10^−6^	*Streptococcus*	19,500	1.29 × 10^−3^
**P3**				*Candida albicans*	214	1.42 × 10^−5^
**P4**	*Klebsiella pneumoniae* (1 colony)	19	1.44 × 10^−6^	*Klebsiella pneumoniae*	19	1.44 × 10^−6^
**P4**				*Enterobacteriaceae*	111	8.40 × 10^−6^
**P4**				*Candida glabrata*	33,336	2.52 × 10^−3^
**P5**	*Klebsiella pneumoniae* (2+)	2,006	2.33 × 10^−3^	*Klebsiella pneumoniae*	2,006	2.33 × 10^−3^
**P5**	*Pseudomonas aeruginosa*(2+)	1,446	1.69 × 10^−3^	*Pseudomonas aeruginosa*	1,446	1.69 × 10^−3^
**P5**	*Citrocbactor freundii*(2+)	20,851	2.42 × 10^−2^	*Enterobacteriaceae*	101,143	1.18 × 10^−1^
**P5**	*Enterococcus faecium* (2+)	40,149	4.67 × 10^−2^	*Enterococcus*	42,463	4.93 × 10^−2^
**P5**	*Candida tropicalis* (small amount)	36	4.18 × 10^−5^	*Candida tropicalis*	36	4.18 × 10^−5^
**P5**				*Klebsiella oxytoca*	29,816	3.47 × 10^−2^
**P5**				*Streptococcus*	83	9.65 × 10^−5^
**P6**	*Streptococcus anginosus* (1+)	2,945	3.22 × 10^−3^	*Streptococcus*	6,812	7.44 × 10^−3^
**P6**	*Serratia marcescens* (3 colonies)	589	6.44 × 10^−4^	*Serratia marcescens*	589	6.44 × 10^−4^
**P6**	*Staphylococcus aureus:MRSA* (1 colony)	17	1.86 × 10^−5^	*Staphylococcus*	531	5.80 × 10^−4^
**P6**	*Prevotella, porphyromonas* (3+)^a^	22,634	3.51 × 10^−4^	*mecA(methicillin-resistancegene)*		
**P6**	*Fusobacterium nucleatum* (3+)^a^	42,998	2.50 × 10^−4^	*Enterobacteriaceae*	67	7.32 × 10^−5^
**P6**	*Peptostreptococcus micros* (3+)^a^	229	4.70 × 10^−2^	*Candida albicans*	0	
**P7**	*Escherichia coli* (3+)	95,875	9.75 × 10^−3^	*Escherichia coli*	95,875	9.75 × 10^−3^
**P7**	*Klebsiella pneumoniae* (3+)	5,455	5.55 × 10^−4^	*Enterobacteriaceae*	114,365	1.16 × 10^−2^
**P7**	*Bacteroides fragilis* group (2+)^a^	8,157	8.30 × 10^−4^	*Klebsiella pneumoniae*	5,455	5.55 × 10^−4^
**P7**	*Eubacterium* sp. (2+)^a^	208	2.12 × 10^−5^	*Klebsiella oxytoca*	2,102	2.14 × 10^−4^
**P7**	*Morganella morganii* (small amount)^a^	295	3.00 × 10^−5^	*Streptococcus*	562	5.72 × 10^−5^
**P7**				*Candida albicans*	5	5.09 × 10^−7^
**P7**				*Candida glabrata*	28	2.85 × 10^−6^
**P8**	*Enterococcus faecalis* (1+)	249	2.58 × 10^−2^	*Enterococcus*	254	2.63 × 10^−2^
**P8**	*Enterococcus avium* (1+)	2	2.07 × 10^−4^	*Candida albicans*	0	0
**P8**	*E.coli* (after 24 h enrichment)	0	0			
**P9**	*Prevotella* sp. (2+)^a^	89	3.72 × 10^−6^	*Streptococcus*	772	3.23 × 10^−5^
**P10**	*Citrobacter freundii* (few colonies)	77	4.12 × 10^−6^	*Enterobacteriaceae*	2,400	1.28 × 10^−4^

a Microorganisms not included in the blood culture identification panel.

#### 3.2.1. Patient 3 (P3)

**Figure 1:**
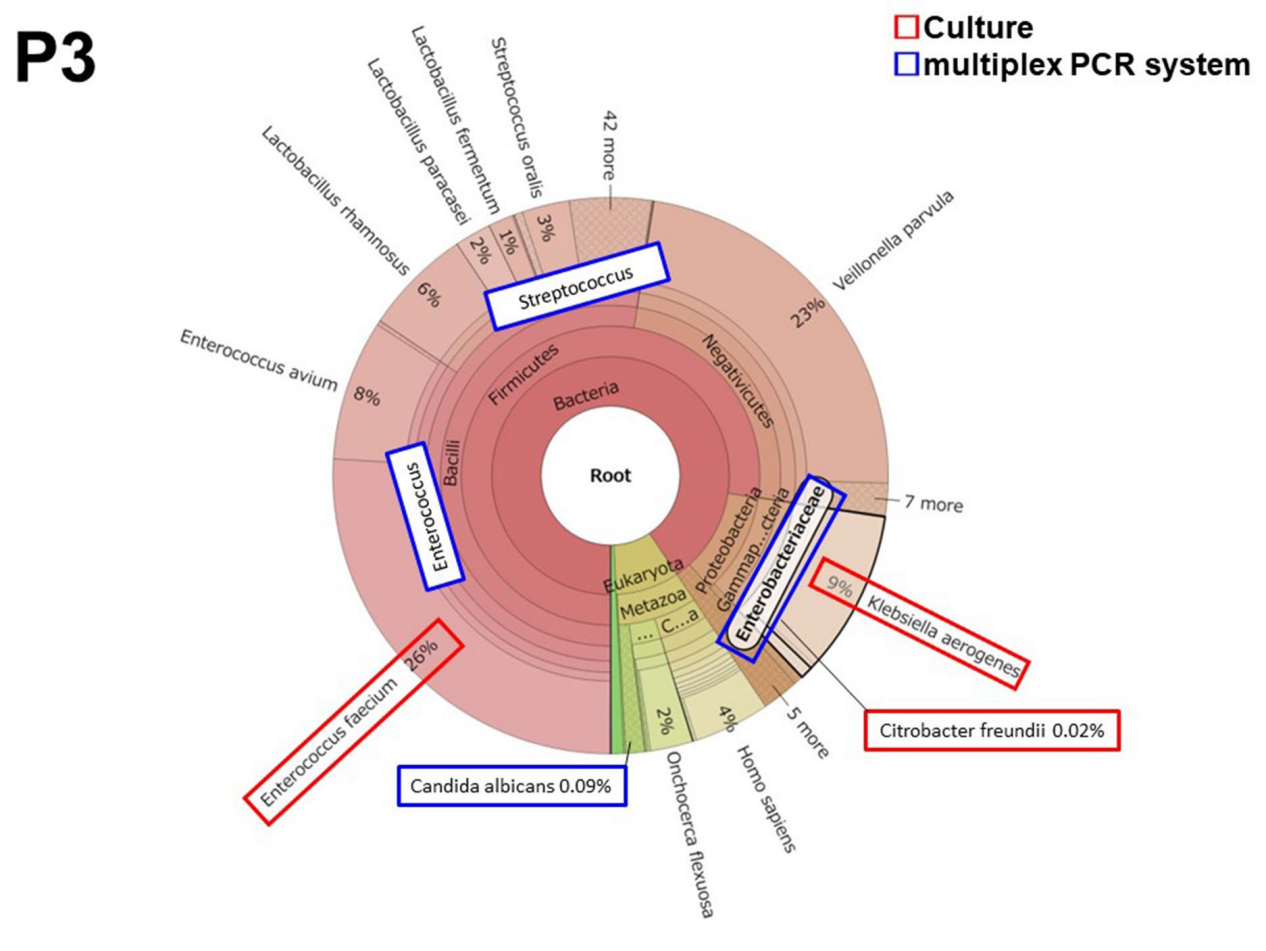
The results of metagenomic analysis Patient 3 (P3).

Patient 3 had peritonitis due to jejunal perforation by double-balloon endoscopy for the treatment of chronic pancreatitis. *Enterococcus* and *Enterobacteriaceae* including *Klebsiella* and *Citrobacter* were detected both in culture and by the multiplex PCR system. Two culture-negative but multiplex PCR system-positive microorganisms were detected in *Streptococcus* spp. (19,500 reads, 1.29 × 10^−3^ of total reads) and in *Candida albicans* (214 reads, 1.42 × 10^−5^ of total reads). All microorganisms were also detected by metagenomic analysis (Figure 1). The metagenomic analysis detected *Enterococcus faecium* and *Enterococcus avium*. However, the multiplex PCR system BCID panel shows “*Enterococcus*,” but the brackets signify that the details of the *Enterococcus* spp. cannot be identified (Supplementary Table S1). Hence, the number of bacterial reads of “*Enterococcus*” in Table 4 was the number of bacterial reads for *Enterococcus faecium* and *Enterococcus avium* combined.

#### 3.2.2. Patient 6 (P6)

**Figure 2:**
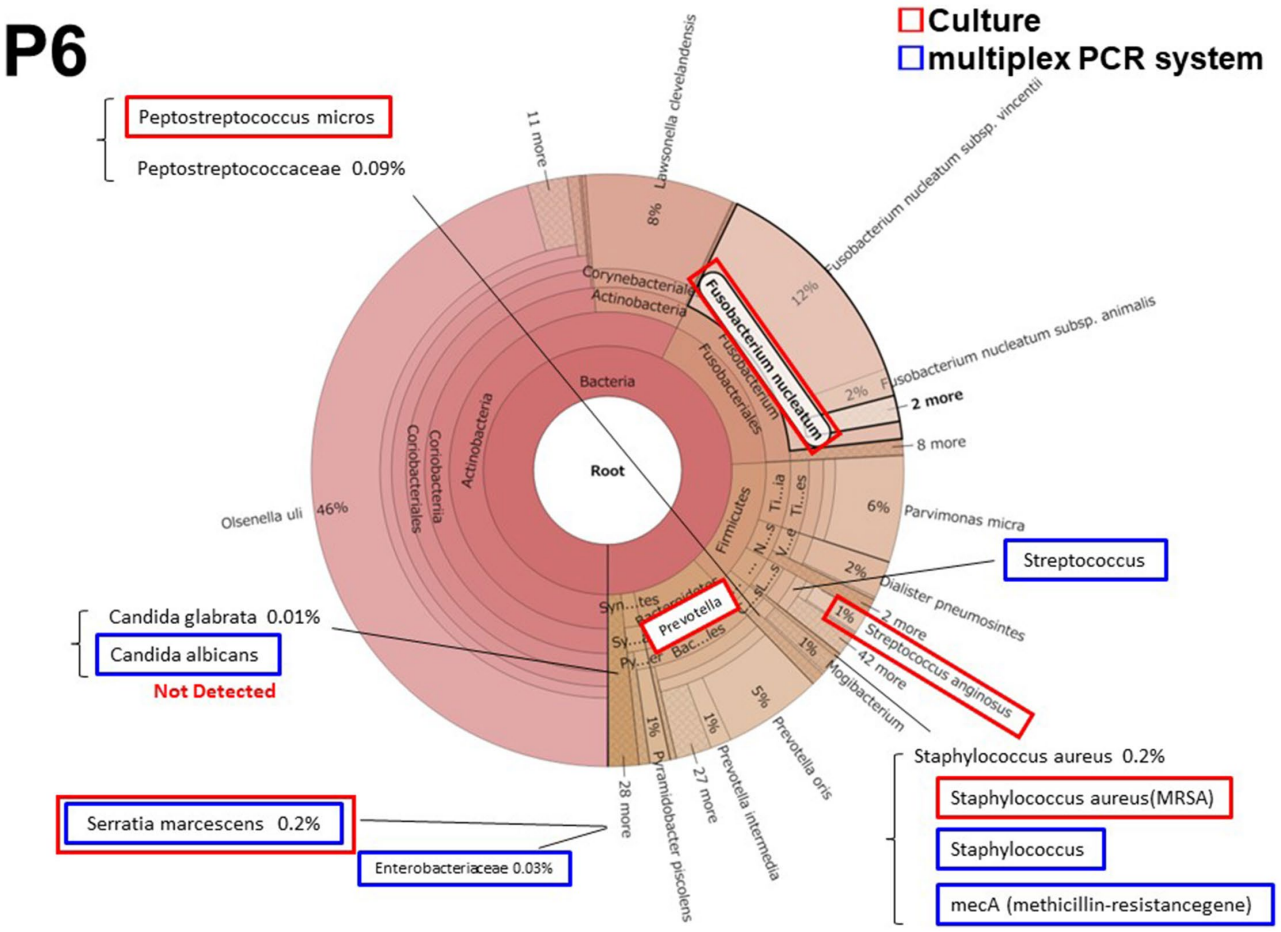
The results of metagenomic analysis Patient 6 (P6).

Patient 6 had a persistent postoperative intraabdominal abscess due to pancreatic fistula after distal pancreatectomy. It was the only cases in which methicillin-resistant *Staphylococcus aureus* (MRSA) was detected by culture and the multiplex PCR system. *Streptococcus* and *Serratia marcescens* were also detected both in culture and by the multiplex PCR system. *Prevotella* and *Fusobacteriales* were predominantly detected both in culture and by metagenomic analysis (Figure 2); however these microorganisms were not included in the multiplex PCR system BCID panel. *Candida albicans* was also detected by the multiplex PCR system. However, the metagenomic analysis detected *Candida glabrata* but not *Candida albicans*.

#### 3.2.3. Patient 7 (P7)

**Figure 3:**
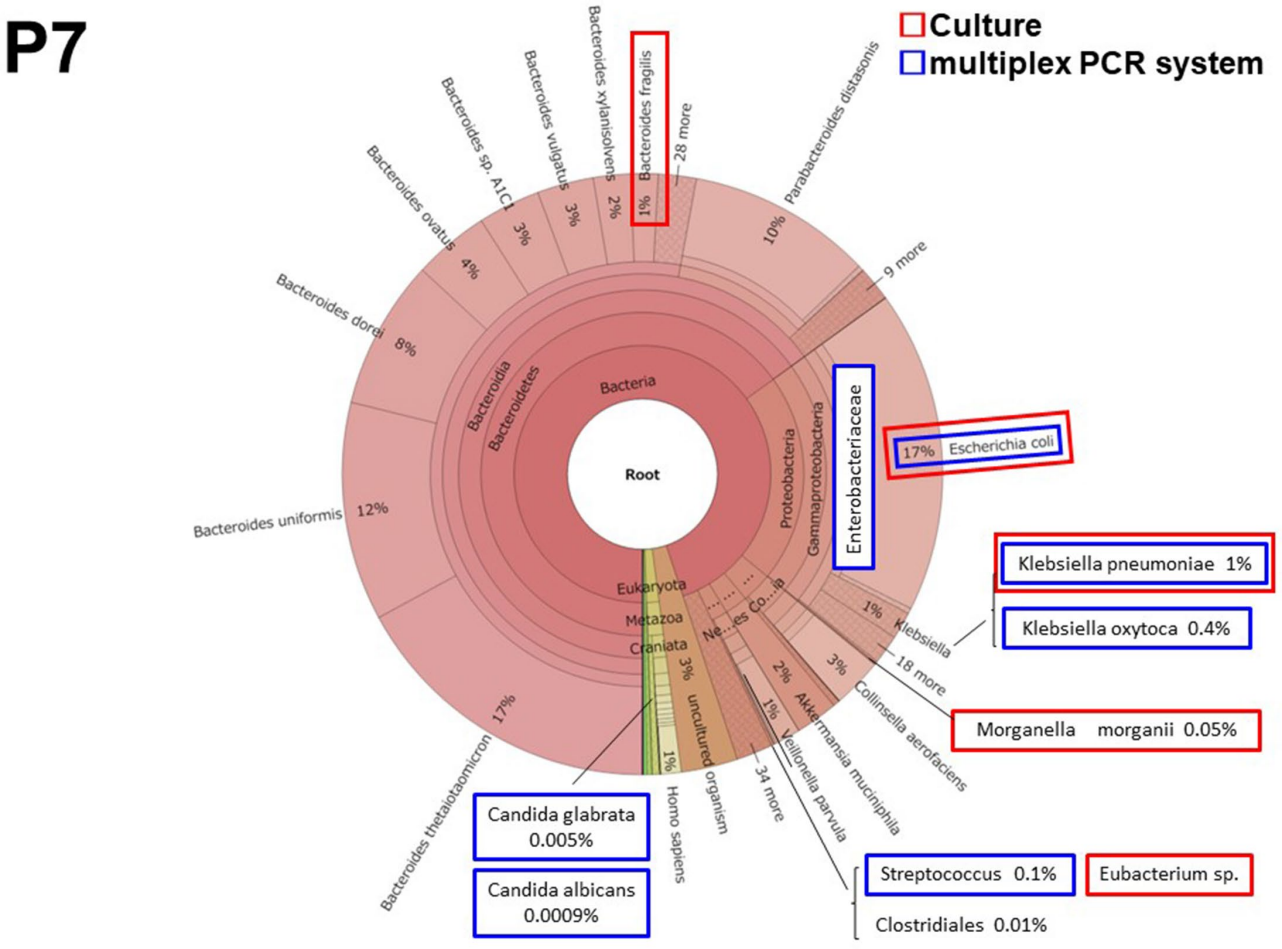
The results of metagenomic analysis Patient 7 (P7).

Patient 7 had perforated peritonitis with septic shock due to rupture of a small intestinal metastatic tumor. It was the most severe and the only fatal infection of the 10 cases. *E. coli* and *Enterobacteriaceae*, including *Klebsiella* spp., were detected both in culture and by the multiplex PCR system (Figure 3). The multiplex PCR system also detected *Candida albicans* in 5 reads (5.09 × 10^−7^ of total reads). This was the lowest number of yeast reads detected using the multiplex PCR system in this study.

## 4. Discussion

In this study, we directly evaluated intraabdominal specimens using the multiplex PCR system. The multiplex PCR system BCID panel detected as high as 90.5% of the microorganisms isolated by culture. We then verified the usefulness of this system and comprehensively evaluated the microorganisms by metagenomic analysis.

Previously, we conducted basic research on rapid identification of causative bacteria of acute biliary tract infection using metagenomic analysis and the Verigene system (Kujiraoka et al., 2017; Watanabe et al., 2021). Use of the Verigene system allowed the identification of causative bacteria within 2 h, but with a detection rate of only 35.7% (Watanabe et al., 2021). We considered that the detection rate may be lower in specimens with small amounts of bacteria (less than 10^6^ CFU/mL of bacteria) or in specimens with multiple bacteria (Watanabe et al., 2021). Moreover, another problem was that Gram staining is necessary in advance because the test panels differ for Gram-positive and -negative bacteria. In this study, we used the multiplex PCR system as a new rapid identification system, which is expected to be more sensitive than the Verigene system (Huang et al., 2016). In this study, multiplex PCR system was able to detect 90.5% of isolated organisms. Further, the multiplex PCR system was able to accurately identify the causative bacteria even when multiple bacteria were present. Furthermore, this system can evaluate Gram-positive and-negative bacteria in the same panel without the need for Gram staining.

The multiplex PCR system is reported to be more sensitive than culture and has a much shorter turnaround time (Timbrook et al., 2021). Rapid identification of the causative bacteria using the multiplex PCR system has been reported to enable the early initiation of appropriate antimicrobial therapy and significantly decreased antimicrobial use and length of hospital stay (Qian et al., 2020; Torres-Miranda et al., 2020; Rule et al., 2021). Rule et al. (2021) and Zheng et al. (2023) also reported that 38% and 58.8% of patients were changed to an appropriate antimicrobial agent by using the rapid identification system for the causative bacteria. Furthermore, mortality associated with inappropriate antimicrobial use was significantly decreased with the use of the multiplex PCR system (Rule et al., 2021). In this way, the multiplex PCR system enables rapid identification of causative bacteria, and as a result, appropriate antimicrobial therapy can be instituted, which is expected to decrease mortality and shorten hospital stay. Limiting the use of broad-spectrum antimicrobial agents may contribute to the prevention of future outbreaks of AMR bacteria.

Multiplex PCR system analysis has been mainly performed on blood samples, spinal fluid, sputum, and feces, and with few reports on intraabdominal abscess and ascites. To date, it has remained unclear whether the multiplex PCR system BCID panel, which is used for blood specimens, can be used to examine for bacteria specific to intraabdominal abscess and ascites, since there is no panel yet for intraabdominal infections. Micó et al. (2015) assessed the multiplex PCR system BCID panel directly on clinical specimens, including ascites and abscess. The panel detected 79% of the microorganisms isolated by culture. In the present study, we also directly evaluated intraabdominal specimens using a multiplex PCR system BCID panel. As a result, all samples except that of P9, which could not be evaluated by the multiplex PCR system BCID panel, had at least one identifiable microorganism. Furthermore, the multiplex PCR system BCID panel detected as high as 90.5% of the microorganisms isolated by culture. According to the metagenomic analysis, the minimum reads count of bacteria detected by the multiplex PCR system was 19 reads of *Klebsiella pneumoniae* in P4. These results suggest that even a small number of bacterial reads could identify the causative bacteria using the multiplex PCR system. On the other hand, some microorganisms such us *Streptococcus* and *Candida* were detected as culture-negative but multiplex PCR system-positive. In addition, *Candida albicans* was detected by the multiplex PCR system in P6. However, the metagenomic analysis detected *Candida glabrata* but not *Candida albicans*. In P8, the multiplex PCR system detected *Candida albicans*, but the metagenomic analysis did not detect *Candida* spp. The reasons for these situations are unclear. The multiplex PCR system might detect not only true causative bacteria but also indigenous bacteria and already dead bacteria. In addition, it might be more likely to be false positive for *Candida albicans*. These unknowns need to be clarified using a large case series.

*E. coli* is an important bacterium in intraabdominal infections. In this study we used three methods for its evaluation: culture, the multiplex PCR system, and metagenomic analysis. All three methods were able to detect *E. coli* in P7, where there was a large number of bacterial reads and where *E. coli* was clinically considered to be the causative bacteria. On the other hand, a small amount of *E. coli* was detected in culture in P1, but could not be detected by the multiplex PCR system, and the number of *E. coli* reads in this patient was 178 reads. P5, which had 522 *E. coli* reads, was not detected by either culture or the multiplex PCR system. This detection failure of such a small number of bacteria with the multiplex PCR system may be not considered a clinical problem, but further evaluation should be made in series of cases to determine the level of bacteria that can be identified.

To our knowledge, our present study is the first to not only compare the results of bacterial culture and the multiplex PCR system, but also to comprehensively prove the detail of the bacterial results using metagenomic analysis. Nevertheless, this study has several limitations. First, it was a retrospective study and only 10 cases were analyzed. The reason for this limitation was the complicated testing procedures and high testing costs hamper the metagenomic analysis with high patient numbers. However, according to the metagenomic analysis, we were able to quantitatively evaluate the bacterial content using the number of bacterial reads as well as the percentage of bacteria. We therefore concluded that the accuracy of the multiplex PCR system is sufficient to allow its use in clinical practice for intraabdominal infections. In the near future, we plan to conduct a comparative study of the multiplex PCR system and bacterial culture using a large case series. Second, regarding the AMR bacteria, only one sample (P6) included MRSA. This sample was accurately evaluated by the multiplex PCR system. In contrast, however, ESBL-producing *Enterobacteriaceae*, although not encountered in this study, are currently a problem in intraabdominal infections (Thompson, 2022), but cannot be detected by the multiplex PCR system BCID panel used in this study. Similarly, this panel cannot distinguish among *Enterococcus* spp., which are frequently detected in acute abdominal infections. These points may also be cited as limitations. Our next large case series will be performed using the multiplex PCR system BCID2 panel, which can also evaluate for ESBL-producing *Enterobacteriaceae* and *Enterococcus* spp. (Supplementary Table S2).

In conclusion, we found that the multiplex PCR system was able to directly evaluate intraabdominal specimens with a high detection rate. The multiplex PCR system may be an affordable rapid identification system for the causative bacteria of acute abdominal infections.

## Data availability statement

The datasets presented in this study can be found in online repositories. The names of the repository/repositories and accession number(s) can be found in the article/Supplementary material.

## Ethics statement

The studies involving human participants were reviewed and approved by the protocol of this study was approved by the Ethics Committee of our hospital (approval number: H21090_H17077) and the National Institute of Infectious Diseases (approval number: 722). The patients/participants provided their written informed consent to participate in this study. Written informed consent was obtained from the individual(s) for the publication of any potentially identifiable images or data included in this article.

## Author contributions

NK, KA, and MkK designed the study concept, revised the manuscript regarding intellectual content, and analyzed data. TS provided technical support. RW, MnK, MK, and HM collected the clinical specimens from patients with acute abdominal infections. MW and YS approved the final draft of this manuscript to be published. All authors contributed to the article and approved the submitted version.

## Funding

This study was supported by a Grant-in-Aid from the Japan Agency for Medical Research and Development (AMED) under grant JP19fk0108048.

## Conflict of interest

The authors declare that the research was conducted in the absence of any commercial or financial relationships that could be construed as a potential conflict of interest.

## Publisher’s note

All claims expressed in this article are solely those of the authors and do not necessarily represent those of their affiliated organizations, or those of the publisher, the editors and the reviewers. Any product that may be evaluated in this article, or claim that may be made by its manufacturer, is not guaranteed or endorsed by the publisher.

## Abbreviations

PCR: polymerase chain reaction
AMR: antimicrobial-resistant
BCID: blood culture identification
