# Supplementary material for: Rapid identification of bacteria using a multiplex polymerase chain reaction system for acute abdominal infections
Source: Front Microbiol. 2023 Jul 10;14:1220651. doi: 10.3389/fmicb.2023.1220651 (PMC10363666; doi:10.3389/fmicb.2023.1220651)
Supplement: Supplementary file 1 [file Table_4.docx]

TABLE 4 | Results of culture and the multiplex PCR system with metagenomic analysis; *Microorganisms not included in the blood culture identification panel”

Sample ID	Culture-positive microorganism	Number of reads in the metagenomic analysis	Rate of reads in the metagenomic analysis	Multiplex PCR system -positive microorganism	Number of reads in the metagenomic analysis	Rate of reads in the metagenomic analysis
**P1**	*Streptococcus anginosus* (2+)	3,723	2.17×10^{-4}	*Streptococcus*	6,796	3.96×10^{-4}
**P1**	*E.coli* (small amount)	178	1.04×10^{-5}			
**P2**	*Cadida albicans* (small amount)	1,905	7.93×10^{-5}	*Candida albicans*	1,905	7.93×10^{-5}
**P3**	*Enterococcus faecium* (1+)	63,060	4.19×10^{-3}	*Enterococcus*	83,555	5.55×10^{-3}
**P3**	*Klebsiella aerogenes* (1+)	23,065	1.53×10^{-3}	*Enterobacteriaceae*	25,387	1.69×10^{-3}
**P3**	*Citrobacter freundii* (small amount)	57	3.78×10^{-6}	*Streptococcus*	19,500	1.29×10^{-3}
**P3**				*Candida albicans*	214	1.42×10^{-5}
**P4**	*Klebsiella pneumoniae* (1 colony)	19	1.44×10^{-6}	*Klebsiella pneumoniae*	19	1.44×10^{-6}
**P4**				*Enterobacteriaceae*	111	8.40×10^{-6}
**P4**				*Candida glabrata*	33,336	2.52×10^{-3}
**P5**	*Klebsiella pneumoniae* (2+)	2,006	2.33×10^{-3}	*Klebsiella pneumoniae*	2,006	2.33×10^{-3}
**P5**	*Pseudomonas aeruginosa*(2+)	1,446	1.69×10^{-3}	*Pseudomonas aeruginosa*	1,446	1.69×10^{-3}
**P5**	*Citrocbactor freundii*(2+)	20,851	2.42×10^{-2}	*Enterobacteriaceae*	101,143	1.18×10^{-1}
**P5**	*Enterococcus faecium* (2+)	40,149	4.67×10^{-2}	*Enterococcus*	42,463	4.93×10^{-2}
**P5**	*Candida tropicalis* (small amount)	36	4.18×10^{-5}	*Candida tropicalis*	36	4.18×10^{-5}
**P5**				*Klebsiella oxytoca*	29,816	3.47×10^{-2}
**P5**				*Streptococcus*	83	9.65×10^{-5}
**P6**	*Streptococcus anginosus* (1+)	2,945	3.22×10^{-3}	*Streptococcus*	6,812	7.44×10^{-3}
**P6**	*Serratia marcescens* (3 colonies)	589	6.44×10^{-4}	*Serratia marcescens*	589	6.44×10^{-4}
**P6**	*Staphylococcus aureus:MRSA* （1 colony)	17	1.86×10^{-5}	*Staphylococcus*	531	5.80×10^{-4}
**P6**	*Prevotella, porphyromonas* (3+)*	22,634	3.51×10^{-4}	*mecA(methicillin-resistancegene)*		
**P6**	*Fusobacterium nucleatum* (3+)*	42,998	2.50×10^{-4}	*Enterobacteriaceae*	67	7.32×10^{-5}
**P6**	*Peptostreptococcus micros* (3+)*	229	4.70×10^{-2}	*Candida albicans*	0	
**P7**	*Escherichia coli* (3+)	95,875	9.75×10^{-3}	*Escherichia coli*	95,875	9.75×10^{-3}
**P7**	*Klebsiella pneumoniae* (3+)	5,455	5.55×10^{-4}	*Enterobacteriaceae*	114,365	1.16×10^{-2}
**P7**	*Bacteroides fragilis* group (2+)*	8,157	8.30×10^{-4}	*Klebsiella pneumoniae*	5,455	5.55×10^{-4}
**P7**	*Eubacterium* sp. (2+)*	208	2.12×10^{-5}	*Klebsiella oxytoca*	2,102	2.14×10^{-4}
**P7**	*Morganella morganii* (small amount)*	295	3.00×10^{-5}	*Streptococcus*	562	5.72×10^{-5}
**P7**				*Candida albicans*	5	5.09×10^{-7}
				Candida glabrata	28	2.85×10^{-6}
**P8**	*Enterococcus faecalis* (1+)	249	2.58×10^{-2}	*Enterococcus*	254	2.63×10^{-2}
**P8**	*Enterococcus avium* (1+)	2	2.07×10^{-4}	*Candida albicans*	0	0
**P8**	*E.coli* (after 24h enrichment)	0	0			
**P9**	*Prevotella* sp. (2+)*	89	3.72×10^{-6}	*Streptococcus*	772	3.23×10^{-5}
**P10**	*Citrobacter freundii* (few colonies)	77	4.12×10^{-6}	*Enterobacteriaceae*	2,400	1.28×10^{-4}
